# Interleukin-22 attenuates renal tubular cells inflammation and fibrosis induced by TGF-β1 through Notch1 signaling pathway

**DOI:** 10.1080/0886022X.2020.1753538

**Published:** 2020-04-25

**Authors:** Rong Tang, Xiangcheng Xiao, Yang Lu, Huihui Li, Qiaoling Zhou, Patrick Kwadwo Nuro-Gyina, Xia Li

**Affiliations:** a Department of Nephrology, Xiangya Hospital, Central South University, Changsha, Hunan, China; b Department of Microbial Infection and Immunity, Ohio State University, Columbus, OH, USA

**Keywords:** TGF-β1, renal tubular cell, inflammation, fibrosis, IL-22, Notch1 pathway

## Abstract

Transforming growth factor-β1 (TGF-β1) is a crucial factor implicated in the development of renal inflammation and tubulointerstitial fibrosis (TIF). The cytokine interleukin 22 (IL-22) was previously reported to involve in the pathogenesis of chronic inflammatory diseases, however recent studies showed that IL-22 could reduced inflammatory responses and tissue damage. In the present study, we aim to investigate the role and mechanisms of IL-22 in renal tubular cells inflammation and fibrosis induced by TGF-β1. HK-2 cells were treated with TGF-β1 in the presence of IL-22 or the Notch pathway inhibitor dibenzazepine (DBZ) for 48 h. Collagen I (Col I), fibronectin (FN), α-smooth muscle actin (α-SMA), vimentin and E-Cadherin were detected by western blot, proinflammatory factors (TNF-α, IL-6) and chemokines (MCP-1, RANTES) were evaluated by ELISA. Jagged1, Notch1, NICD1, and Hes1 were also detected by western blot. We found TGF-β1 increased the levels of Col I, FN, α-SMA and vimentin in HK-2 cells compared with control, and decreased E-Cadherin level, however, IL-22 restored their expressions partly. IL-22 reduced overexpression of proinflammatory factors (TNF-α, IL-6) and chemokines (MCP-1, RANTES) levels induced by TGF-β1, along with down-regulation of Jagged1, Notch, NICD1 and Hes1. Fibrosis and inflammation in renal tubular cells induced by TGF-β1 could be attenuated by IL-22, and the effects were similar to DBZ treatment. Collectively, our study shows that IL-22 exerts a protective role in renal fibrotic and inflammatory responses induced by TGF-β1 *in vitro*, which may be through inhibiting Jagged1/Notch1 signaling pathway activation.

## Introduction

Although glomerular diseases are main causes of chronic kidney diseases (CKD), renal tubulointerstitial fibrosis (TIF) is a common final pathological process underlying the progression of various kidney diseases, which leads to end-stage renal disease (ESRD) [1]. Pathological analysis shows that renal function damage and prognosis correlates better with the extent of tubulointerstitial impairment than with the degree of glomerular damage [2]. The proximal tubular cell is a type of important cell during the development and progression of renal TIF [3]. In addition, among various mechanisms, renal inflammation plays a prominent role in the progression of renal TIF. Multiple factors are involved in renal inflammation, including cytokines, chemokines, and growth factors [4–6]. Particularly, transforming growth factor-β1 (TGF-β1) has been described as the core inducer of renal inflammation and fibrogenic responses, mediating infiltration of inflammatory cells, epithelial-mesenchymal transition (EMT) of tubular epithelial cells, and deposition of extracellular matrix (ECM) [7–9]. Thus, the regulation of fibrosis mediated by TGF-β1 in the kidney is of great interest.

Recent studies have shown that Notch signaling emerges as contributors to the progression of kidney fibrosis [10,11]. Notch signaling pathway is highly conserved in mammals, which consists of at least 4 Notch receptors (Notch 1–4) and 5 Notch ligands (Delta-like l, 3, 4 and Jagged 1, 2) in vertebrates. Following ligand binding, Notch receptor, a transmembrane protein, undergoes enzymatic proteolysis by the γ-secretase complex subsequently, resulting in cytoplasmic release of Notch intracellular domain (NICD). Then NICD translocates into the nucleus where it forms a complex with the DNA-binding protein CSL (RBP-Jk/CBF1), leading to the transcription of notch target genes, such as Hes1 and Hey1 [12,13]. There are mounting evidences that TGF-β1 activates Jagged1/Notch1 signaling pathway in renal epithelial cells, then promotes development of renal TIF [14,15]. However, the molecular mechanisms involved in this process remain unexplored.

Interleukin-22 (IL-22), a member of IL-10 cytokine family, has been reported to exert either protective or pathogenic effects in the development of autoimmune and inflammatory diseases, depending on different types of microenvironment [16–19]. Recent studies suggest that IL-22 suppresses renal damage in acute kidney injury and CKD by sustaining tubular epithelial integrity [20,21]. Although these studies demonstrate the roles of IL-22 in protection of kidney diseases, up to now, the effects and mechanisms of IL-22 in renal TIF have not been confirmed.

In this study, we conducted an *in vitro* experiment to explore the effects of exogenous recombinant IL-22 on inflammation and fibrosis of human renal tubular epithelial cells treated by TGF-β1, and to investigate the underlying mechanisms of IL-22 in this process. We were intrigued to explore whether IL-22 could exert protective effects against renal inflammatory response and fibrosis through inhibiting Notch1 pathway activation induced by TGF-β1 *in vitro*.

## Materials and methods

### Cell culture

Human tubular epithelial cells (HK-2) from American Type Culture Collection (ATCC, Manassas, VA) were cultivated in DMEM/F12 medium (Hyclone, USA) supplemented with 10% fetal bovine serum (FBS, Gibco, USA) in a humidified 5% CO2 incubator at 37 °C [22]. When reached 80% confluence, the cells were trypsinized using 0.05% trypsin with 0.02% EDTA, then subcultured by a 1:3 split ratio in a new flask.

### Cell treatments

HK-2 cells were seeded in the condition as described above, then cultured in serum-deprived medium and incubate for 24 h prior to usage. Recombinant human TGF-β1 (PeproTech, Rocky Hill, NJ, USA), IL-22 (ProSpec, East Brunswick, NJ, USA), the γ-secretase inhibitor dibenzazepine (DBZ) (Syncom, Groningen, Netherlands) were used in our experiments. Cultured HK-2 cells were treated with increasing concentrations (10–40 ng/ml) of IL-22 for 48 h, then appropriate concentration of IL-22 was added to cells for different times (24–96 h), the cell proliferation and cytotoxicity were detected respectively. Prior to stimulation with TGF-β1 (10 ng/ml) as decribed previously [23], one group of cell was exposed to the γ-secretase inhibitor DBZ (1 μM) which can inhibit signaling from all Notch receptor type as described [24]. Cells were randomly divided into the following groups: (1) control group; (2) TGF-β1 group, treated by 10 ng/ml TGF-β1 for 48 h; (3) TGF-β1 + IL-22 group, treated by 10 ng/ml TGF-β1 and 20 ng/ml IL-22 for 48 h; (4) TGF-β1 + DBZ group, HK-2 cells were pretreated with 1 μM DBZ for 1 h, then stimulated with 10 ng/ml of TGF-β1 for 48 h. After incubation, cells were arranged for further experiments. Each experiment was repeated at least three times.

### Cell proliferation assay

We used MTT assay as described previously [25] to detect the proliferation of HK-2 cells affected by IL-22 or DBZ. HK-2 cells of logarithmic growth period were seeded in the FBS free medium in a sterile 96-well plate at a density of 1 × 10^4^ cells/well for 12 h, then medium was exchanged to 100 µl medium containing 2% FBS. Different concentrations of IL-22 (10, 20, 30 and 40 ng/ml) were added to HK-2 cells for 48 h. Based on the results of above experiment, HK-2 cells were treated with IL-22 (20 ng/ml) for different times (24, 48, 72 and 96 h). HK-2 cells were incubated alone with different doses of DBZ (0.1, 0.5, 1, 2, and 5 μM) for 48 h. After incubation, 5 mg/ml MTT (Sigma, New York, NY, USA) 20 µl was added to each well. After incubation at 37 °C for 4 h, the supernatants were carefully removed, 150 µl dimethyl sulfoxide (DMSO) was added into each well and shaken for 10 min to dissolve the formazan. After the precipitation was dissolved completely, the optical density (OD) was measured by a 96-well plate reader at 570 nm.

### Cytotoxicity assay

To determine the cytotoxicity effect of IL-22 on HK-2 cells, we evaluated cell membrane integrity by levels of lactate dehydrogenase (LDH) in the culture media as described [26], since LDH was delivered from the cytosol of damaged cells. After the intervention of IL-22 (10, 20, 30 and 40 ng/ml) for 48 h, or treatment of IL-22 (20 ng/ml) for different times (24, 48, 72 and 96 h), the cytotoxicity of HK-2 cells was determined by the Pierce LDH Cytotoxicity Assay Kit (Thermo Scientific, Waltham, MA, USA) according to the manufacturer’s instructions. LDH activity was normalized to protein concentration and results were evaluated as fold of controls.

### Western blot and immunopreciptation analysis

HK-2 cells were divided into four groups: control, TGF-β1, TGF-β1 + IL-22, and TGF-β1 + DBZ. Adherent cells were collected and lysed for 30 min with ice-cold RIPA buffer, and supernatant was harvested after centrifugation at 12,000 g at 4 °C for 20 min. Cell lysate (about 50 µg) was boiled at 95 °C for 5 min in loading buffer, separated by SDS-PAGE, next transferred onto a 0.45 μm nitrocellulose membrane (Millipore, Billerica, MA, USA). Nonspecific immunoreactive sites on the membranes were blocked with 5% defatted milk for 1 h at room temperature. The membrane was incubated with primary antibodies overnight at 4 °C. The primary antibodies were as follows: anti-Notch1, anti-Jagged1, anti-Hes1, and anti-collagen I (Col I) antibodies were from Abcam (Cambridge, MA, USA). Anti-NICD2, anti-IL-22RA1, anti-NICD1, anti-CSL (RBPJK), anti-phospho-Smad3 (pSmad3) and anti-Smad3 antibodies were from Abcam, too. Anti-fibronectin (FN), anti-α-smooth muscle actin (α-SMA), anti-vimentin, anti-E-Cadherin, anti-NICD4, and anti-β-actin antibodies were from Sigma(New York, NY, USA). Anti-NICD3 antibody was from Cell Signaling (Danvers, MA, USA). After several washes, the membrane was then incubated with IgG horse-radish peroxides conjugate secondary antibody at room temperature for 1 h. The levels of protein expression were normalized to β-actin levels and quantified using ECL chemiluminescence system (Thermo Scientific Pierce, Shanghai, China). For co-immunoprecipitation (Co-IP) as previously described [27], HK-2 cells were collected and lysed with 0.5% NP40 lysis buffer. The cell lysates were immunoprecipitated with anti-IL-22R1 antibody, then blotted with anti-Notch1 or anti-IL-22R1 antibody. The films were scanned and the intensity of the bands was determined by Quantity One software (Bio-rad, Hercules, CA, USA).

### Enzyme-linked immunosorbent assay (ELISA)

After the incubation time, the supernatant was collected, centrifuged and then used for further detection. TNF-α, IL-6 and MCP-1 levels in the supernatant were assessed by using ELISA kits (Ebioscience, San Diego, CA, USA), RANTES concentration was evaluated by ELISA kits (R&D System, Minneapolis, MN, USA), according to the manufacturer’s instructions. The optical density of each well was quantified by using an ELISA reader at 450 nm. If wavelength correction was available, set to 570 nm or 620 nm.

### Statistical analysis

The data were expressed as the mean ± standard deviation (SD). The data from different groups were compared using one-way analysis of variance (ANOVA), followed by LSD test. Statistical analyses were performed using SPSS 19.0 Statistical software (Chicago, IL, USA), and *p* value < .05 were considered statistically significant.

## Results

### Effects of IL-22 on the viability and cytotoxicity of HK-2 cells

**Figure 1.**
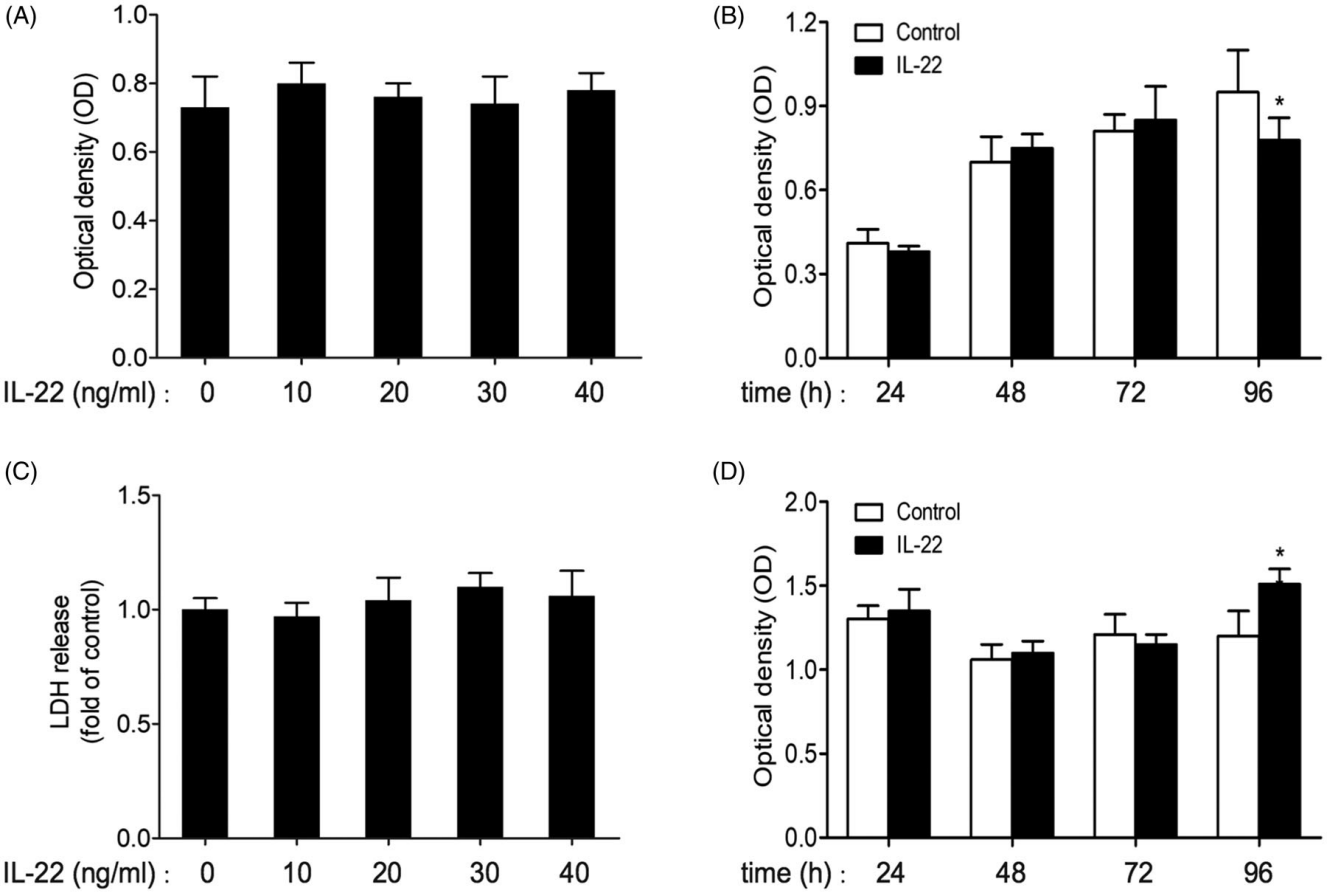
Effects of IL-22 on the viability and cytotoxicity of HK-2 cells. (A) Viability of HK-2 cells treated with different concentrations of IL-22 (10–40 ng/ml) for 48 h was detected by MTT assay. (B) Viability of HK-2 cells stimulated with IL-22 (20 ng/ml) for different times (24–96 h) was assessed *via* MTT assay. (C) HK-2 cells were treated with different doses of IL-22 (10–40 ng/ml) for 48 h, and cytotoxicity was evaluated by LDH assay. (D) HK-2 cells were intervened with IL-22 (20 ng/ml) for increasing times (24–96 h), then cytotoxicity was evaluated by LDH assay. *p <.05, compared with control group at the same time point.

In order to evaluate effects of IL-22 on the proliferation and cytotoxicity of HK-2 cells, we performed MTT and LDH assays in the culture media. As shown in Figure 1(A), MTT assay suggested that different concentrations of IL-22 (10, 20, 30, 40 ng/ml) alone had no effect on viability of HK-2 cells after incubation for 48 h (*p* > .05). Combined with the result of western blot, IL-22 (20 ng/ml) was chosen to incubate with HK-2 cells for different time periods (24, 48, 72, 96 h). Compared to control of the same time point, IL-22 exerted no obvious effect on cell viability during 24–72 h, however, the cell proliferation was reduced in the group of 96 h (*p <* .05; Figure 1(B)).

LDH assay showed that IL-22 (10–40 ng/ml) did not affect LDH release level when incubated with cells for 48 h (*p* > .05; Figure 1(C)). These results indicated that IL-22 (10–40 ng/ml) treatment for 48 h displayed no apparent influence on proliferation and cytotoxicity of HK-2 cells. Similarly, as shown in Figure 1(D), LDH release was increased in the time period of 96 h intervened by IL-22 (20 ng/ml) compared with control (*p <* .01), and kept unchanged at other time points (24–72 h). Thus, IL-22 (20 ng/ml) incubation for 48 h was chosen for subsequent experiment.

We also detected whether increasing concentrations of DBZ (0.1, 0.5, 1, 2, 5 μM) treatment alone for 48 h influenced cell proliferation. Compared with control, DBZ (0.1–2 μM) did not affect cell viability (*p* > .05). Cell viability of 5 μM DBZ group was lower than control (*p <* .01, Supplementary Figure 1). Therefore, 1 μM DBZ as described [24] was used for subsequent study.

### Effects of different doses and times of IL-22 on Notch1 pathway induced by TGF-β1 in HK-2 cells

**Figure 2.**
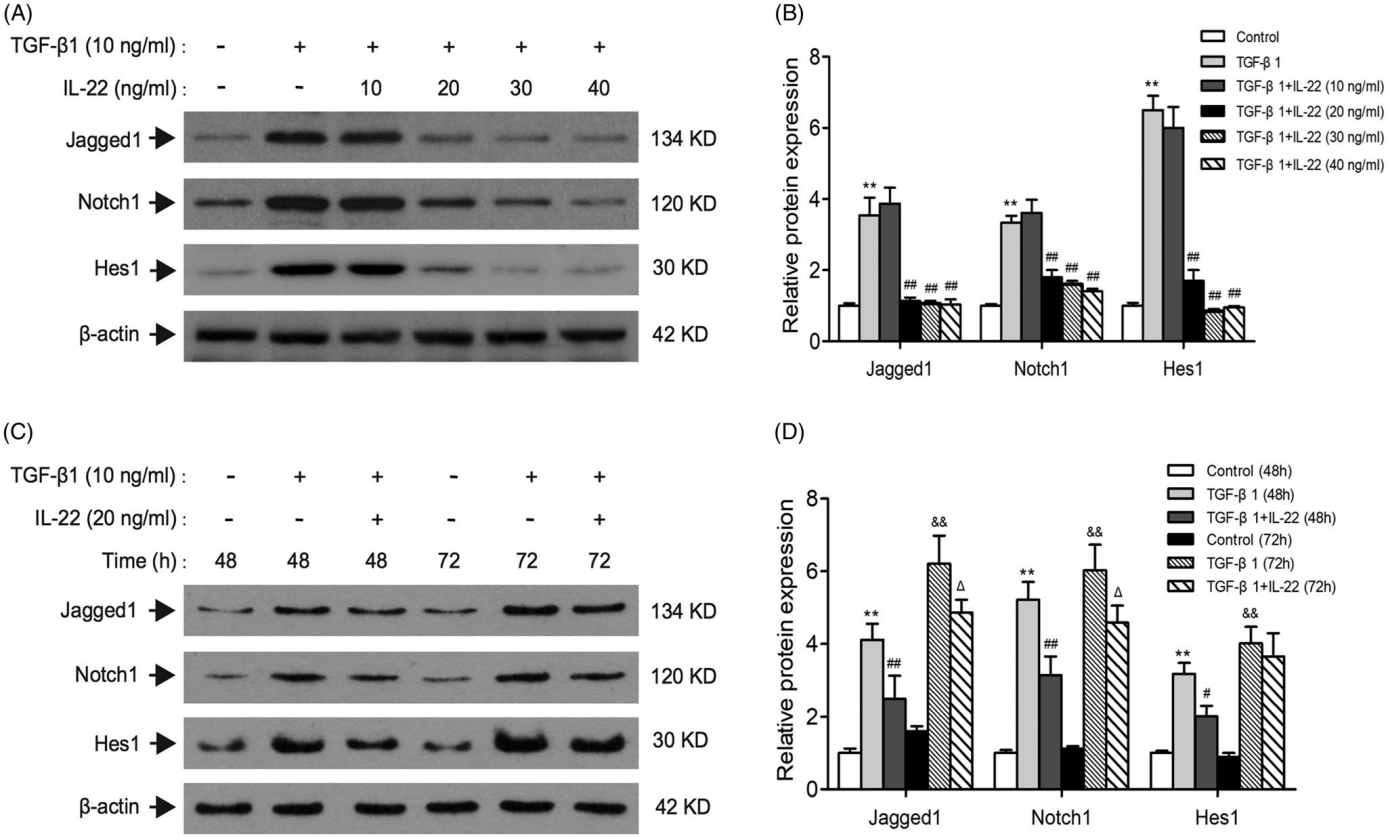
Effects of different doses and times of IL-22 on Notch1 pathway expression stimulated by TGF-β1 in HK-2 cells. (A) Western blot of Jagged1, Notch1, and Hes1 expressions in HK-2 cells treated with TGF-β1 in the presence of different doses of IL-22 (10–40 ng/ml) for 48 h. (C) Western blot of Jagged1, Notch1, and Hes1 expressions in HK-2 cells stimulated by TGF-β1 in the presence of IL-22 (20 ng/ml) for different times (24–96 h). (B) Quantitative analysis of western blot shown in A. **p <.01, compared with control group; ^##^*p* <.01, compared with TGF-β1 group. (D) Quantitative analysis of western blot shown in C. **p < .01, compared with control group (48 h), ^#^*p* <.05, ^##^*p* <.01, compared with TGF-β1 group (48 h); ^&&^*p* <.01, compared with control group (72 h), ^Δ^*p* <.05, compared with TGF-β1 group (72 h).

Previous studies have indicated that TGF-β1 treatment significantly increase Jag1 and Notch1 expressions in culture of renal tubular epithelial cells, revealing that TGF-β1 is an upstream modulator of Notch signaling [14,15]. To determine whether IL-22 could relieve the activation of Notch1 pathway induced by TGF-β1, we detected crucial proteins of Notch1 pathway in the presence of IL-22 (10–40 ng/ml) with TGF-β1 for 48 h in HK-2 cells. Results showed in Figure 2(A) revealed that TGF-β1 upregulated Jagged1, Notch1, and Hes1 markedly after 48 h (*p* < .01). IL-22 at the dose of 10 ng/ml showed no obvious effect on Jagged1, Notch1 and Hes1 expressions compared with TGF-β1 group (*p* > .05). However, IL-22 (20–40 ng/ml) decreased levels of Jagged1, Notch1 and Hes1 protein induced by TGF-β1 (*p <* .01). This effect was similar among the groups of 20 ng/ml, 30 ng/ml and 40 ng/ml IL-22 (*p* > .05). Subsequently, IL-22 (20 ng/ml) was added to cells treated by TGF-β1 at 48 h and 72 h respectively, the levels of Jagged1, notch1, and Hes1 were detected by western blot. We found IL-22 (20 ng/ml) treatment for 48 h exerted the greatest inhibitory effects on Jagged1/notch1 signaling in our study (Figure 2(C)). Therefore, we used 20 ng/ml IL-22 incubation with cells for 48 h in the following experiments.

### IL-22 reduced inflammation of HK-2 cells induced by TGF-β1

**Figure 3.**
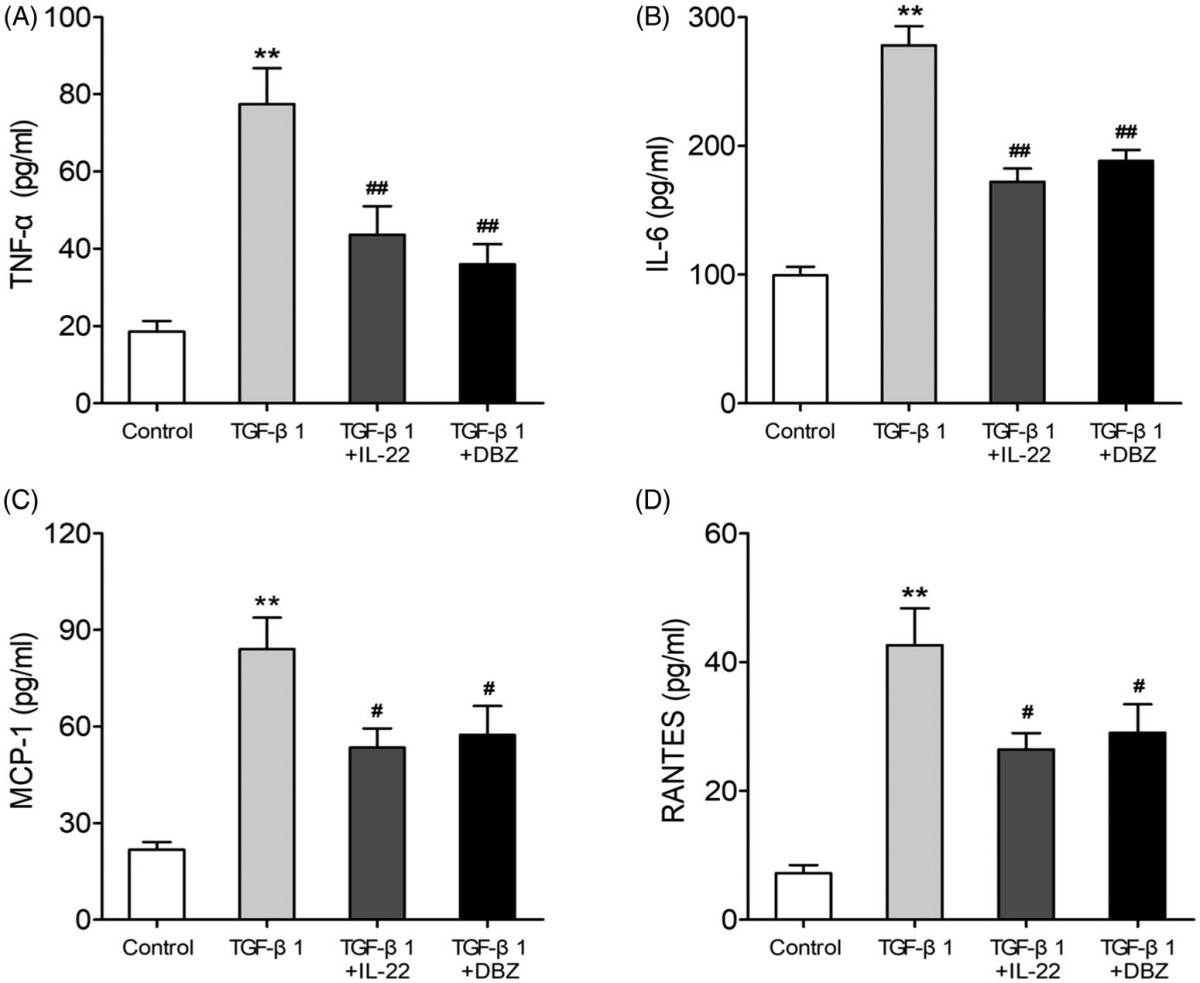
IL-22 reduced proinflammatory factors (TNF-α, IL-6) and chemokines (MCP-1, RANTES) levels in HK-2 cells induced by TGF-β1. HK-2 cells were divided into four groups including control, TGF-β1, TGF-β1 + IL-22 and TGF-β1 + DBZ, the expressions of above cytokines in supernatant of HK-2 cells were assessed *via* ELISA. (A) The level of TNF-α in four groups. (B) The concentration of IL-6 in four groups. (C) The concentration of MCP-1 in four groups. (D) The level of RANTES in four groups. **p <.01, compared with control group; ^#^*p* <.05, ^##^*p* <.01, compared with TGF-β1 group.

Renal inflammation plays a major role in the development of renal TIF, and many factors are involved in this process including cytokines, chemokines and growth factors [6]. To explore the effects of IL-22 on inflammation of HK-2 cells stimulated by TGF-β1, we examined the proinflammatory factors (TNF-α, IL-6) and chemokines (MCP-1, RANTES) levels by ELISA. As shown in Figure 3, compared with control, TGF-β1 elevated TNF-α (77.45 ± 10.38 pg/ml vs. 18.63 ± 3.01 pg/ml), IL-6 (278.05 ± 16.73 pg/ml vs. 99.19 ± 7.61 pg/ml), MCP-1 (84.25 ± 11.23 pg/mlvs. 21.79 ± 3.11 pg/ml) and RANTES (42.66 ± 6.55 pg/ml vs. 7.24 ± 1.53 pg/ml) in HK-2 cells (*p* < .01). The concentrations of proinflammatory cytokines TNF-α (43.65 ± 8.31 pg/ml or 36.07 ± 5.74 pg/ml) and IL-6 (171.98 ± 11.41 pg/ml or 188.12 ± 10.15 pg/ml) were lower in TGF-β1 + IL-22 or TGF-β1 + DBZ group than TGF-β1 group (*p* < .01), and the levels of chemokines MCP-1 (57.23 ± 9.14 or 54.61 ± 7.05) and RANTES (26.48 ± 2.89 pg/mlor 29.14 ± 5.11 pg/ml) were decreased, too (*p* < .05). There were no significant differences in these indexes between the groups of TGF-β1 + IL-22 and TGF-β1 + DBZ IL-22 (*p* > .05). These data indicated that IL-22 could reduce inflammation of HK-2 cells treated by TGF-β1.

### IL-22 reduced fibrosis of HK-2 cells induced by TGF-β1

**Figure 4.**
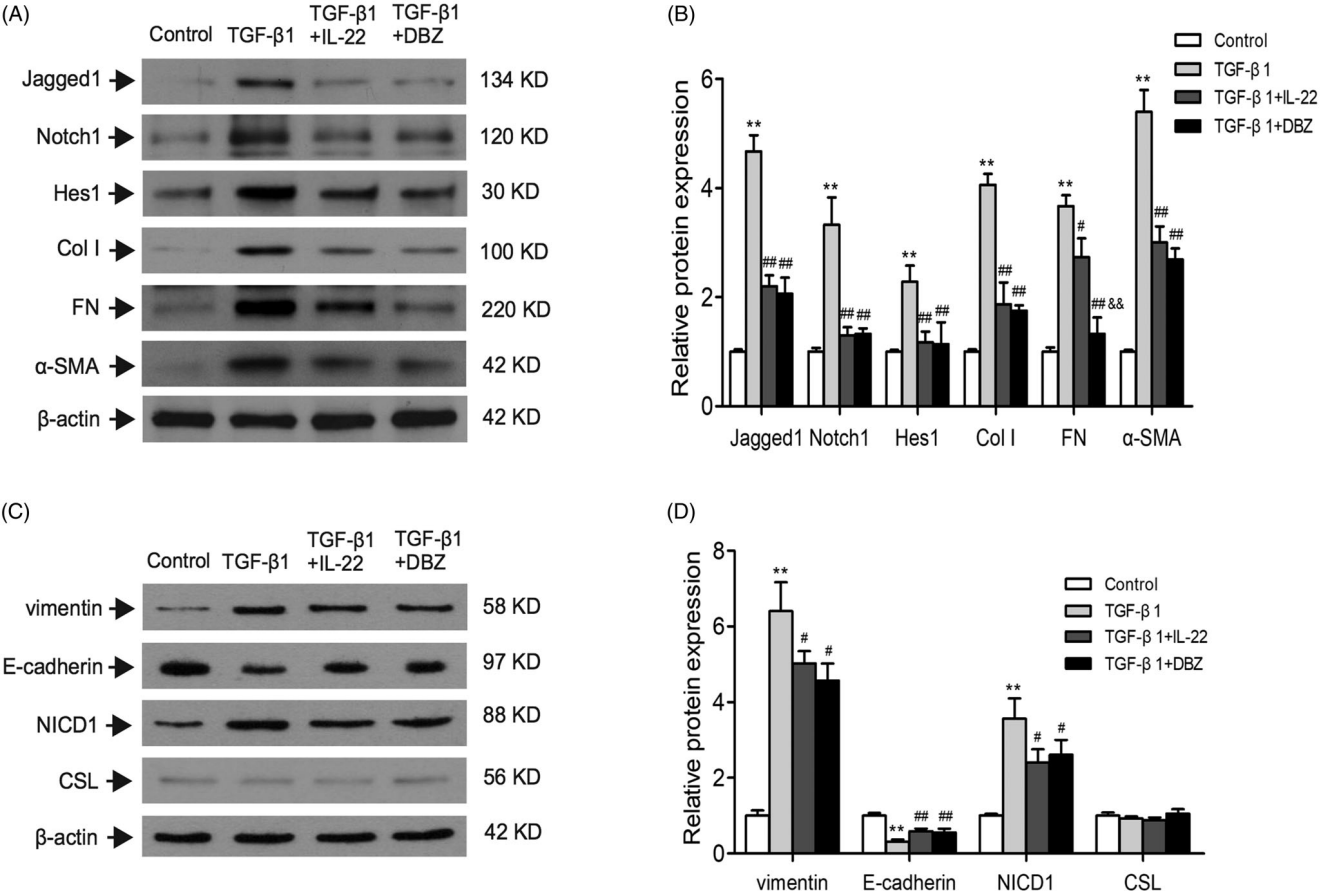
IL-22 reduced Notch1 pathway activation and fibrosis in HK-2 cells induced by TGF-β1. HK-2 cells were divided into four groups including control, TGF-β1, TGF-β1 + IL-22, and TGF-β1 + DBZ, the related protein levels in HK-2 cells were detected by western blot. (A) Western blot analysis of Jagged1, Notch1, Hes1, Col I, FN, and α-SMA expressions in HK-2 cells. (C) Expressions of vimentin, E-cadherin, NICD1, and CSL were determined by western blot. (B, D) Quantitative analysis of protein expressions shown in A and C. **p <.01, compared with control group; ^#^*p* <.05, ^##^*p*<.01, compared with TGF-β1 group; ^&&^*p* < .01, compared with TGF-β1 + IL-22 group.

TGF-β1 is considered as a crucial mediator of EMT process in renal tubular cells, thus contributes to the progression of renal fibrosis. During EMT, the renal epithelial cells undergo the stepwise loss of epithelial proteins such as E-cadherin, and the acquisition of mesenchymal markers such as α-SMA and vimentin [7,8]. In order to determine the effect of IL-22 on fibrosis of HK-2 cells treated by TGF-β1, western blot was conducted to assess EMT related proteins levels such as α-SMA, vimentin, and E-cadherin. Also, we evaluated the expressions of fibrosis-related protein such as Col I and FN. As shown in Figure 4, the levels of Col I, FN, α-SMA, and vimentin were significantly increased in TGF-β1 group compared with control, but E-cadherin was downregulated (*p* < .01). The group of TGF-β1 + IL-22 or TGF-β1 + DBZ partly restored their expressions (*p* < .05 or *p* < .01). The level of FN was lower in group of TGF-β1 + DBZ than TGF-β1 + IL-22 (*p* < .01). There were no differences in other indexes between TGF-β1 + IL-2cf2 and TGF-β1 + DBZ groups (*p* > .05). These data suggested that IL-22 reduced fibrosis of HK-2 cells induced by TGF-β1.

### IL-22 might reduce inflammation and fibrosis through suppressing Notch1 pathway activation in HK-2 cells treated by TGF-β1

Next, we sought to determine whether IL-22 diminished renal tubular cells inflammation and fibrosis *via* inhibiting Notch1 pathway activation induced by TGF-β1. Western blot revealed that there were increased protein expressions of Jagged1, Notch1, Hes1 and NICD1 in TGF-β1 group (*p* < .01), and their expressions were downregulated in group of TGF-β1 + IL-22 or TGF-β1 + DBZ (*p* < .01); there were no obvious differences between the two groups (*p* > .05). There was no significant difference in CSL expression among control, TGF-β1, TGF-β1 + IL-22 and TGF-β1 + DBZ groups (*p* > .05; Figure 4 (A,C)).

**Figure 5.**
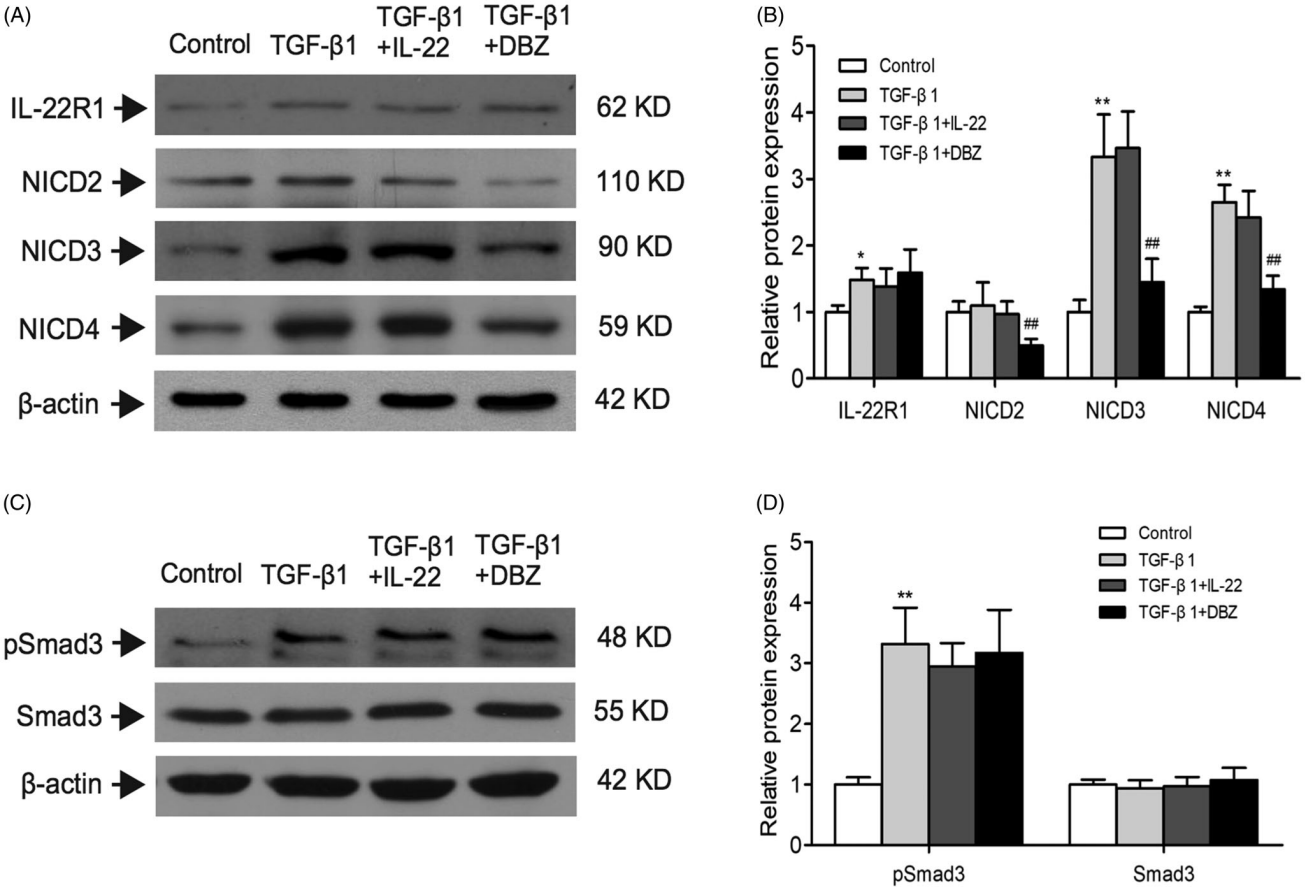
Effects of IL-22 on the expressions of IL-22R1, NICD2–4, and pSmad3 in HK-2 cells treated with TGF-β1. These protein expressions were evaluated by western blot in control, TGF-β1, TGF-β1 + IL-22, and TGF-β1 + DBZ groups, (A) Western blot analysis of IL-22R1, NICD2, NICD3, and NICD4 levels in HK-2 cells. (C) Expressions of pSmad3 and Smad3 were detected by western blot. (B, D) Quantitative analysis of protein expressions shown in A and C. *p <.05, **p <.01, compared with control group; ^##^*p*<.01, compared with TGF-β1 group.

IL-22 exerts its biological effects *via* binding to a heterodimeric transmembrane receptor complex composed of IL-22R1 and IL-10R2, and subsequent activation of signaling pathways. IL-10R2 has been shown to be ubiquitously expressed. IL-22R1 expression is restricted to epithelial cells and determines whether a cell is an IL-22 target or not [28]. We detected the level of IL-22R1 in HK-2 cells, and found that TGF-β1 group displayed higher level of IL-22R1 than control (*p* < .01). Whereas IL-22R1 expression was similar among TGF-β1, TGF-β1 + IL-22, and TGF-β1 + DBZ groups (*p* > .05; Figure 5(A)). In order to explore whether IL-22R1 interacted with Notch1 to conduct the signal pathway activation, we performed Co-IP experiment between the two proteins. However, the result showed that there was no significant interaction between IL-22R1 and Notch1 (Supplementary Figure 2). There might be other molecular mechanisms in this process.

These data supported the hypothesis that IL-22 reduced Notch1 pathway activation induced by TGF-β1 in HK-2 cells.

### Effects of IL-22 on expressions of NICD2–4 and pSmad3 in four groups of HK-2 cells

As you know, Notch signaling includes four types of notch receptor members Notch1, Notch2, Notch3 and Notch4 in mammal. NICD1, NICD2, NICD3, and NICD4 are active forms of Notch receptors [12,13]. Except NICD1, did IL-22 affect other three types of Notch pathway *via* inhibiting the level of NICD? In this regard, we detected NICD2, NICD3 and NICD4 expressions by western blot in HK-2 cells. Figure 5(A) showed that NICD3 and NICD4 were upregulated by TGF-β1, and downregulated after treatment by DBZ (*p* < .01), however IL-22 did not affected them (*p* > .05). Similar to the study performed by Morrissey et al. [14], we found TGF-β1 alone or TGF-β1 combined with IL-22 exerted no obvious effect on the level of NICD2 (*p* > .05), which was decreased in TGF-β1 + DBZ group (*p* < .01). These findings furtherly suggested that IL-22 exerted protective effects through inhibiting Notch1 pathway activation, but not other Notch pathways in HK-2 cells stimulated by TGF-β1.

As TGF-β1/Smad3 has been considered as a major pathway for renal fibrogenesis [29], we detected protein expressions of Smad3 and phospho-Smad3. Result showed Smad3 phosphorylation increased following TGF-β1 treatment compared with untreated control (*p* < .01). Interestingly, TGF-β1 + DBZ did not interfere with level of phospho-Smad3, which was consistent with the study reported previously [30]. Similarly, IL-22 had no obvious effect on the upregulated phosphorylation of Smad3 in HK-2 cells intervened by TGF-β1 (*p* > .05). There was no significant difference in Smad3 level among four groups (*p* > .05; Figure 5(C)). This result indicated IL-22 might not exert its protective effect through inhibiting TGF-β1/Smad3 signaling.

## Discussion

We had hypothesized that the exogenous recombinant IL-22 had reno-protective effects in renal epithelial tubular cells stimulated by TGF-β1 *in vitro*. Indeed, the present study indicated that IL-22 reduced inflammation and fibrosis induced by TGF-β1 in human renal tubular cells and exerted protective effects in this process. Notably, our data reported for the first time that IL-22 might relieve the renal tubular cells injury induced by TGF-β1 *in vitro via* suppression of Notch1 pathway, and IL-22 might be a new therapeutic target.

It has been shown that TGF-β1 is a critical cytokine that initiates and promotes renal tubulointerstitial inflammation and fibrosis. Substantial evidence has confirmed that TGF-β1 induces inflammatory cytokines production in the proximal tubules, and mediates fibrotic response in kidneys [7,8]. Consistent with the previous findings [23], we used TGF-β1 at the concentration of 20 ng/ml to stimulate tubular cells for 48 h, and found that TGF-β1 elevated concentrations of proinflammatory cytokines (TNF-α and IL-6) and chemokines (MCP-1 and RNATES), and also induced overexpression of fibrotic molecules such as Col I, FN, α-SMA, and vimentin indicating that TGF-β1 promoted the inflammation and fibrosis *in vitro* (Figures 3 and 4).

It has been shown that, upon both acute and chronic renal injury, leukocytes are recruited to the injured kidneys and secret cytokines to regulate inflammation and fibrosis [31]. Among them, cytokine IL-22 has been proven to be involved in these process [32]. IL-22 is mainly secreted by lymphoid cells such as Th22, Th17, NKT and innate lymphoid cells (ILCs) [32], Th22. Recent studies have reported the reno-protective effects of IL-22 in acute kidney injury and CKD [20,21], however we and other groups verified that Th22 and IL-22 promoted renal damage in IgA nephropathy and hypertension [33–36]. Whether IL-22 is protective or destructive in kidney diseases may depend on different microenvironment. IL-22 sustains epithelial integrity in progressive kidney remodeling and fibrosis during acute kidney injury [20]. Wang et al. [37] observe that IL-22 exerts anti-fibrotic effects in diabetic nephropathy *via* reducing renal NLRP3/caspase-1/IL-1β pathway. Therefore, except acute kidney injury, Il-22 can also play a protective role in chronic kidney disease. Thus, our study was in line with the previous study which suggested IL-22 might exert reno-protective effects during certain condition. As shown in Figures 3 and 4, IL-22 not only reduced fibrotic molecules expressions such as Col I, FN, α-SMA, and vimentin, increased epithelial protein E-cadherin strikingly, but also decreased the levels of inflammatory cytokines including TNF-α, IL-6, MCP-1 and RANTES in tubular cells stimulated by TGF-β1, thus, further unveiling the biological function of IL-22 in this process. These results revealed IL-22 inhibited fibrosis and inflammation of renal tubular cells induced by TGF-β1. However, IL-22 did not affect increased level of pSmad3, suggesting IL-22 might not exert its protective effect *via* suppressing TGF-β1/Smad3 signaling (Figure 5).

IL-22 is secreted by immune cells and can binds to a membrane receptor complex IL-22R, which is a heterodimeric receptor composed of IL-22R1 and IL-10R2 [30,38]. IL-10R2 is extensively expressed in various cells, and IL-22R1 expression is restricted to epithelial cells of non-hematopoietic origin, including skin, gut, lung and kidney, but deficiency on immune cells. IL-22 and IL-22R thus can form a type of ‘immune-epithelial’ signaling, resulting in the activation of downstream signaling pathways [39,40]. In the current study, exogenous IL-22 played protective effects in renal tubular cells treated by TGF-β1, and the biological function exerted by IL-22 might be achieved through binding to IL-22R in renal tubular cells. As shown in Figure 5, we found that TGF-β1 induced higher level of IL-22R1 than control. Whereas IL-22 or DBZ exerted no significant effect on IL-22R1 level increased by TGF-β1. In order to find the underlining molecular mechanisms of IL-22, we detected the effect of IL-22 on classical pathway Notch1 signaling. Interestingly, we found that IL-22 inhibited inflammation and fibrosis, might be through suppressing Notch1/Jagged1 pathway which was confined in renal tubular cells, and this might be a new molecular mechanism of IL-22 in this process.

Further, In order to check whether IL-22 affected other three types of Notch receptors *via* inhibiting expression of NICD, we also detected active form of Notch 2–4 receptors including NICD2, NICD3 and NICD4. We found that NICD3 and NICD4 were upregulated by TGF-β1, NICD2 level was unchanged. IL-22 did not affect NICD2, NICD3 and NICD4 expressions (Figure 5). This result displayed IL-22 might not exert its protective effects through affecting Notch 2–4 activation in HK-2 cells treated by TGF-β1.

Evidences have been shown that Notch1 pathway activation in renal tubular epithelial cells is considered to be both necessary and important for the development of renal inflammation and renal TIF [14,15]. Morrissey et al. [14] find that TGF-β1 induces Jagged1 (a Notch ligand) and it receptor Notch1 expression in fibrotic disease using cultured human renal cortical epithelial cells and mice of unilateral ureteral obstruction (UUO) model, which may be a critical mechanism in this process. Pharmacologic inhibition of Notch1 pathway activation using a γ-secretase inhibitor ameliorates kidney injury [10,40]. Consistently, we found that treatment of cultured HK-2 cells with TGF-β1 upregulated the levels of Jagged1, Notch1, and NICD1, as well as the target molecular Hes1 and downstream inflammatory and fibrotic processes, indicating TGF-β1 stimulation activated the Notch1 signaling pathway. Moreover, this response could be reduced by an inhibitor of γ-secretase, an enzyme required for Notch receptor cleavage and downstream transcription regulation. Our data also showed that IL-22 reduced the Jagged1, Notch1, NICD1, and Hes1 expressions, and downregulated the levels of fibrosis and inflammation-related molecules. The effect was similar to r-secretase inhibitor DBZ (Figure 4). These results indicated that IL-22 ameliorated renal tubular cells inflammation and fibrosis induced by TGF-β1, these effects might be through inhibiting activation of Notch1 pathway *in vitro*.

However, to date, the relationship and underling mechanism between IL-22 and Notch1 signaling pathway, and the exact role of IL-22 signaling pathway in the progression of renal fibrosis are not elucidated. Moreover, it is unknown that the effect of IL-22 signaling is through inhibiting Notch1 signaling directly or indirectly. Whether there are other mechanisms during this process? Also the animal study needs to be added to increase evidence for the vitro study. Further studies are required to explore the effect and mechanism of IL-22 *in vitro* and *in vivo*.

In summary, our study for the first time indicated that exogenous administration of IL-22 exerted a protective effect in inflammation and fibrosis of renal tubular cells induced by TGF-β1, and might be through inhibiting Notch1 signaling pathway activation. The current study demonstrated the protective role of IL-22 in the pathogenesis of tubular cells fibrosis and inflammation, making IL-22 to serve as a novel therapeutic target.

## Supplementary Material

Supplemental Material

Supplemental Material
