# Supplementary figures and images for: Interleukin-22 attenuates renal tubular cells inflammation and fibrosis induced by TGF-β1 through Notch1 signaling pathway
Source: Ren Fail. 2020 Apr 25;42(1):381–90. doi: 10.1080/0886022X.2020.1753538 (PMC7241524; doi:10.1080/0886022X.2020.1753538)

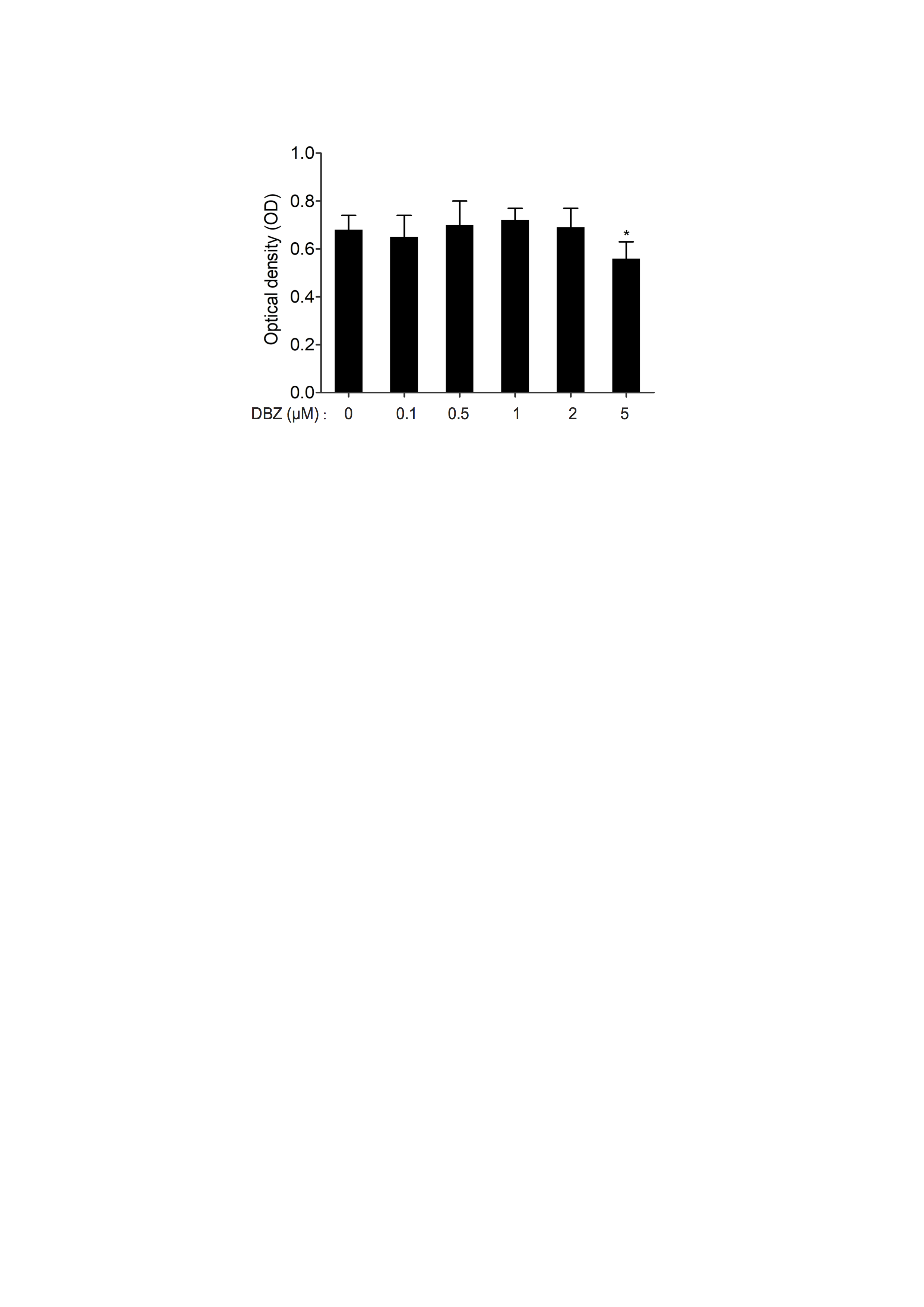

Supplement: Supplemental Material [file IRNF_A_1753538_SM8437.tiff]

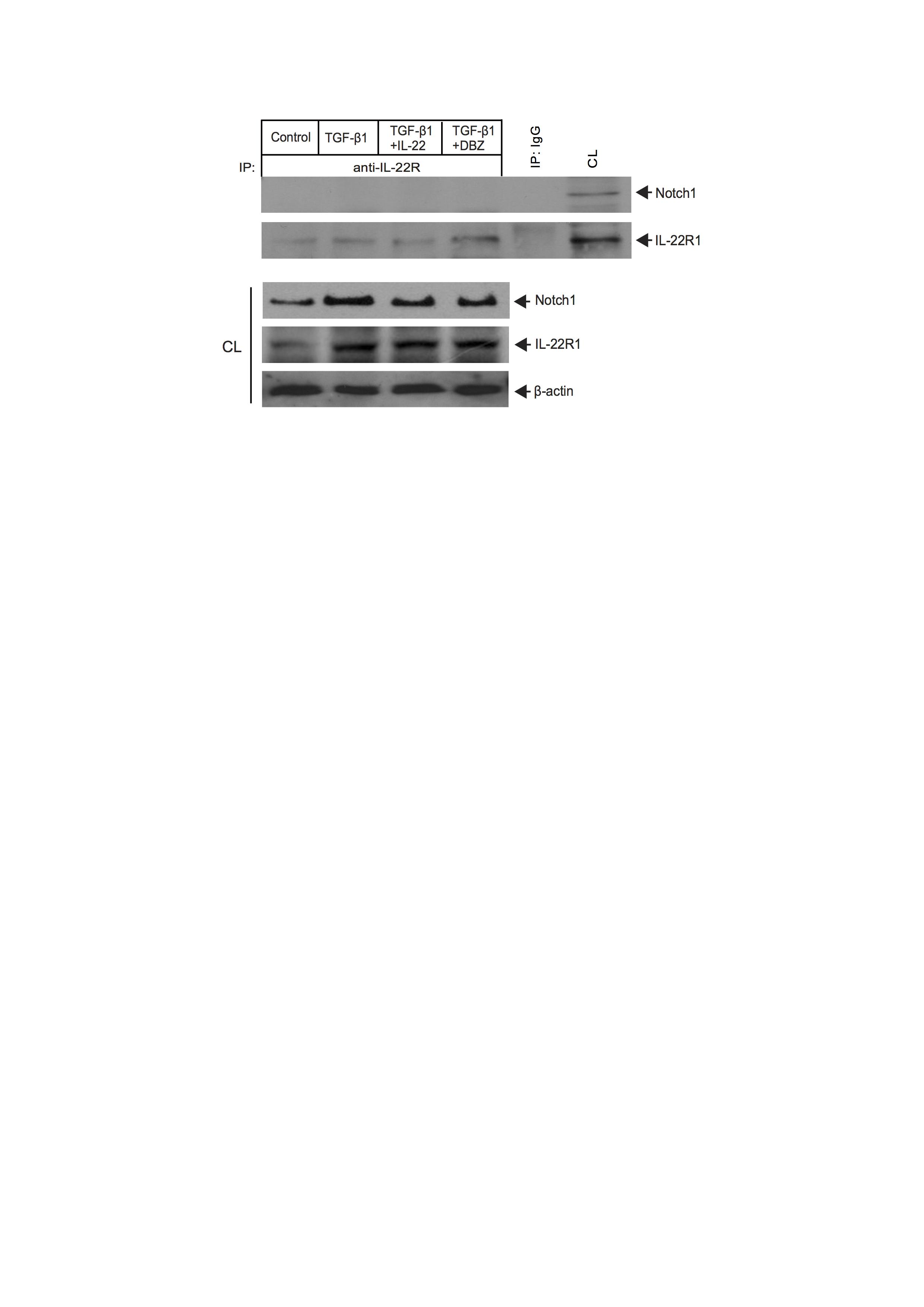

Supplement: Supplemental Material [file IRNF_A_1753538_SM7893.tiff]
